# Enhancing Impact: A Call to Action for Equitable Implementation Science

**DOI:** 10.1007/s11121-023-01589-z

**Published:** 2023-10-25

**Authors:** Rachel C. Shelton, Ross C. Brownson

**Affiliations:** 1 Mailman School of Public Health, Department of Sociomedical Sciences, Columbia University, 722 W 168th Street, New York, NY 10032 USA; 2 Columbia University, Irving Institute for Clinical and Translational Research, New York, NY 10032 USA; 3 Prevention Research Center, Brown School at Washington University in St. Louis, 1 Brookings Drive, Campus, Box 1196, St. Louis, MO 63130 USA; 4 Department of Surgery, Division of Public Health Sciences, and Alvin J. Siteman Cancer Center, Washington University School of Medicine, Washington University in St. Louis, St. Louis, MO 63130 USA

**Keywords:** Implementation, Dissemination, Health equity, Public health, Prevention, Health disparities, Implementation science

## Abstract

Despite investments in evidence-based interventions and Implementation Science, most evidence-based interventions are not widely or routinely adopted, delivered, or sustained in many real-world community and healthcare settings. This gap is even greater in settings and populations experiencing numerous social and structural barriers to health, with important implications for persistent patterns in health inequities. In this Viewpoint, as part of a Special Issue on *Advancing the Adaptability of Chronic Disease Prevention and Management through Implementation Science*, we outline seven calls to action for the field of Implementation Science, with the goal of encouraging researchers, practitioners, and funders to be more intentional and accountable in applying Implementation Science to have greater impact on promoting health equity. Calls to action include (1) enhance public health, community, and multi-sectoral partnerships to promote health equity and equitable implementation; (2) revisit and build the evidence base needed to promote health equity and impact at multiple levels; (3) prioritize focus on policy development, dissemination, and implementation; (4) be agile and responsive in application of Implementation Science frameworks, processes, and methods; (5) identify and redefine meaningful metrics for equity and impact; (6) disseminate scientific evidence and research to a diverse range of partners and potential beneficiaries; and (7) extend focus on de-implementation, mis-implementation, and sustainability which are central to enhancing health equity. Additionally, we outline why a focus on prevention and public health is essential to making progress towards health equity in Implementation Science, summarize important advancements that the field has made towards making equity more foundational, and pose important research questions to enhance equitable impact of work in this area.

There has been significant growth of the field of dissemination and implementation science (hereafter referred to as “implementation science” or IS). IS is the study of methods, strategies, frameworks to actively promote the routine adoption, use, and sustainability of EBIs in real-world clinical, community, and public health settings to improve quality of care and health (Brownson et al., 2023). There are many reasons to be optimistic about the impact that IS can have in making progress towards addressing the ubiquitous gap between research and practice. However, there have also been challenges in realizing the full impact of IS (Beidas et al., 2022) and questions as to whether the science has been applied to benefit and reach systemically marginalized communities and settings that would benefit the most. Additionally, while IS is well-poised to address persistent health inequities and promote health equity, until recently, this has not been an explicit focus of the field, its underlying foundations, or commonly used frameworks or approaches. Further, there have been concerns about the unintended exacerbation of inequities through the conduct of implementation research that does not typically reflect or represent communities experiencing numerous, intersecting structural barriers to health (e.g., more resourced clinics are more likely to have higher organizational “readiness” and infrastructure to support their participation in implementation research; Brownson et al., 2021).

To address these challenges and enhance the promise and impact of IS, we believe it is essential for the field to have a foundational and explicit focus on health equity. Health equity is defined here as “the principle underlying a commitment to reduce, and ultimately, eliminate disparities in health and in its determinants, including social determinants” (Braveman, 2014, p. 6). A focus on health equity recognizes the role of complex historical and ongoing structural and socioeconomic drivers in creating and reinforcing unjust health inequities; for example, structural drivers like racism, sexism, and classism (e.g., often through policies, laws) shape inequitable access to social resources, power, and opportunities (e.g., unequal pay or opportunities for work and education, inequities in wealth, unequal access to safe neighborhoods/housing and quality healthcare; National Academies of Sciences Engineering & Medicine, 2017). Such drivers have critical implications for creating and maintaining health advantages or disadvantages across diverse social groups/identifies (e.g., by race, gender, income). Creating the conditions for health equity requires providing all groups access to the opportunities and resources they need for optimal health. To make progress towards health equity in IS, we encourage greater recognition of the broader context in which health inequities are shaped, prioritization of prevention and policy, as well as stronger bridges between public health, clinical (e.g., community-oriented primary care), and community partners, settings, and practitioners in our implementation efforts. Here, we outline calls to action for the field, particularly in the context of prevention and public health, which we hope will encourage researchers, practitioners, policymakers and funders to be more intentional and accountable in explicitly applying IS to achieve beneficial and meaningful impacts on health equity.

## Advancing the Impact of Implementation Science: Prevention, Public Health, and Health Equity

Investments and prioritization in prevention are essential to making progress towards health equity for numerous reasons. First, early life social and physical contexts lay the foundation for accumulating health promoting or health hindering exposures, resources, and opportunities that have consequences across the lifespan (Braveman & Gottlieb, 2014). Early experiences and environments have fundamental and long-lasting consequences for health, many of which have generational impacts (e.g., poverty, lack of wealth, residential segregation; Cohen & Lê-Scherban, 2015). Investing in the development and implementation of EBIs and policies early in life when trajectories are more malleable and prevention is critical and possible, particularly in communities that have been systemically disadvantaged across generations, is an equity-promoting approach that IS can prioritize.

To make greater progress towards equitable impacts at the population health level, it is essential that the field of IS move beyond a focus on healthcare equity and towards a more comprehensive, multi-sector focus. There is well-placed mistrust of healthcare settings and providers in many communities, and many minoritized groups may not have access to or may not actively seek healthcare in clinical settings (or may actively avoid it) due to stigma, mistrust, discrimination, or other negative experiences in accessing or receiving healthcare (Jaiswal & Halkitis, 2019). Thus, as discussed below, healthcare equity in IS is important but insufficient to fully promote health equity.

Prioritizing a health equity focus in IS requires including and prioritizing public health and community settings (e.g., churches, schools, worksites, departments of health; Mazzucca et al., 2021) for implementation and learning from both implementation failures and successes in these settings. Community-based settings may have greater trust and reach from minoritized communities than healthcare, providing opportunities for making progress towards equity by engaging and investing in existing community leadership, assets, and strengths. IS conducted in partnership with local communities and focused on community and public health settings has strong potential to target root causes of health inequities which must be addressed to promote health equity and equitable implementation (Mensah et al., 2018; Shelton et al., 2023). This includes structural drivers (e.g., structural racism) and social determinants of health (SDOH) (e.g., neighborhood deprivation, economic and educational opportunities) which drive inequities and the “research-to-practice-gap.”

## Progress in Applying Implementation Science to Promote Equity

We applaud the growing transformation in IS and focus on promoting health equity. Equity focused IS has been defined as “ …when strong equity components – including explicit attention to the culture, history, values, assets and needs of the community– are integrated into the principles, strategies, frameworks, and tools of IS” *(*Loper et al., 2021*, p. 4)* and when EBIs that are designed or adapted to promote equity and address inequities and their root causes are routinely implemented in settings serving systemically marginalized communities (Baumann & Cabassa, 2020; Loper et al., 2021). There have been contributions in this area in IS over the past 5 years that we believe will pave the way for more impactful advancements. For example, there are equity-focused conceptual frameworks that can be used to assess contextual determinants and drivers relevant to equity at the patient, provider, and system levels (e.g., discrimination, stigma; Woodward et al., 2019). There have been adaptations to existing IS frameworks that inform understanding of how forms of power operate across implementation phases and how to track implementation outcomes with an explicit equity focus (Baumann & Cabassa, 2020; Stanton et al., 2022). Additionally, there are recommendations for considering how structural racism shapes implementation, how an anti-racism approach can be applied in IS (Shelton et al., 2021; Shelton et al., 2021), and how to select methods that center scientific and health equity (McNulty et al., 2019).

## Calls to Action: Enhancing the Impact of Equitable Implementation Science

Despite progress, important overarching questions remain in enhancing the equitable impact of IS. How can we leverage and expand public health workforce roles to center equity? How can we build infrastructure to support the adoption of EBIs and strengthen systems of care to reduce health inequities, particularly amidst historical underinvestment in public health systems and the need for rebuilding after COVID-19? How can we address SDOH and increase health equity through public health interventions that can be more rapidly scaled and spread? Here, we address calls to action to further health equity in IS and provide key questions related to each (Table 1). As researchers trained in public health and IS, our goal is to further explicate priority areas for researchers, funders, and practitioners that we see as having high potential for impact in advancing equity through IS that have not been fully articulated or realized, particularly in the context of prevention and public health.

**Table 1:** Priority questions and considerations for the field to enhance the equitable impact of Implementation Science

**1. Enhance and Extend Public Health, Community and Multi-sectoral Partnerships to Promote Health Equity & Equitable Implementation**	• How can we better build the trust and trustworthiness of our institutions to facilitate community engagement? • How can we identify and support trusted messengers who can help bridge and synergize community and clinical implementation efforts? • What is the balance of meaningful engagement that is fair, equitable, and does not burnout partners? • What are implementation strategies that can build community capacity, power, resources, and ownership? • How can we overcome the paradox of innovation and disconnect between research to practice to more rapidly learn bi-directionally from community and practice innovations? • What do nexus and bridges need to look like to support more equitable impact in public health and prevention?
**2. Revisit, Build, and Re-imagine the Evidence Base Needed to Promote Health Equity and Impact Multiple Levels**	• How do we take into consideration other factors beyond implementation with a health equity focus? • How can implementing settings and practitioners balance health needs with social needs in systemically marginalized settings? • How can we address underlying structural factors and social determinants of health that affect implementation? • How can we more rapidly build an evidence base to promote equity with EBIs that can be spread and scaled for greater impact? • What are the values, biases, and assumptions we bring (as individuals and in our scientific disciplines) that have implications for the evidence-base, the selection/prioritization of EBIs, and potential unintended consequences? • What are the key types of adaptations to EBIs that matter for enhancing health equity and/or equitable implementation (while still retaining key components linked to effectiveness)? • How can we optimize benefits to populations experiencing unjust and unfair outcomes? • How can we ensure that costs are captured as part of the evidence generation process and in implementation activities so that we can understand the return on investment, cost savings, and budget impact of delivering equity-focused programs and policies?
**3. Prioritize and Elevate a Focus on Policy Development, Dissemination, & Implementation Central to Addressing Equity**	• How can we better develop and enact policies that are aligned with evidence that promotes health equity? • How do we more fully develop policies with early input from those affected by the policies? • How can we better accelerate the widespread dissemination and adoption of equity-focused policies and programs? • How can we optimize and enhance the equitable roll out, reach, and sustainability of policies to maximize health benefits and health equity? • What are some of the unintended consequences of policies that may contribute to or reinforce health inequities? • How can we dismantle and remove policies that are disproportionately harmful to marginalized communities and settings but are entrenched and adaptive in our systems and institutions?
**4. Be Agile, Responsive and Adaptive in Application of Frameworks, Processes, and Methods to Enhance the Impact of Implementation Science**	• How soon should we act on evidence in systemically marginalized communities, even as it is not “perfect” and continues to evolve? • How can we speed the translation of evidence to practice in complex, dynamic, and under-resourced settings? • How can we best measure the pace of research translation in a way that denotes impact? • Does designing EBIs for low-income communities in relation to community needs from the start accelerate speed of uptake over adapting EBIs designed for higher resource contexts and settings? • How can we be more agile and adaptive in learning from both implementation successes and failures in community-based settings and public health settings, to meaningfully reduce health inequities?
**5. Identify and Redefine Meaningful Metrics for Equity & Impact in Implementation Science**	• What do equitable processes look like in implementation science and how do we track them? • What are meaningful shared indicators and validated measures for equity that are pragmatic for the field? • What types of impacts are most meaningful in the context of health equity? • Is a set of implementation strategies more effective in reducing inequities or promoting equity in implementation outcomes? • What are the mechanisms by which implementation strategies impact implementation outcomes across different sub-groups? • What implementation strategies are feasible, acceptable, appropriate for populations experiencing inequities? • How to adapt implementation strategies for organizations serving populations facing structural barriers, to achieve equity in implementation outcomes?
**6. Disseminate Scientific Evidence and Research Findings to Diverse Stakeholders and Partners**	• How best might we involve diverse and underrepresented partners early in designing for dissemination efforts? • How can we facilitate widespread and equitable dissemination for a range of types of settings, partners, and cultures? • How can we apply advancements from the science of dissemination and communication to enhance the reach and impact of research findings for a wider range of partners, practitioners, and communities? • How can we ensure that research products and findings reach systemically marginalized communities and settings? • How can we best communicate equity-focused evidence and policies to a range of systems and contexts with variable resources, to influence evidence adoption and use?
**7. Extend Focus on De-implementation, Mis-implementation, & Sustainability which are Central to Equity**	• What is the prevalence of de-implementation and mis-implementation in lower resource settings and groups? • How do de-implementation and mis-implementation determinants vary in higher and lower resource settings? • For settings experiencing health inequities, how do we best enhance and communicate about underutilization of preventive services while reducing overuse of low-value care? • What implementation and sustainability strategies enhance long-term delivery and transform long-standing patterns of inequities over time? • What determinants matter most for long-term sustainment in low-resources settings and should be prioritized? How are those similar and different than those that matter for implementation? • How can we engage diverse partners in planning for sustainability? • What is the return on investment and broader impact of sustaining preventive and public health interventions, particularly in communities that experience health inequities and settings that have been disinvested?

## Enhance and Extend Public Health, Community, and Multi-sectoral Partnerships to Promote Health Equity and Equitable Implementation

There has been a long history of work outside of IS on the value of community engagement and community-based participatory research (CBPR) to ground research and help ensure its relevance, appropriateness, and impact (Mensah et al., 2018; Wallerstein & Duran, 2010). In the context of IS, there has been growing awareness of the critical importance of co-creation, community engagement, and participatory IS to help lay the foundation for infrastructure and processes to support health equity and equitable implementation (Pérez Jolles et al., 2022; Ramanadhan et al., 2018). While its application has varied in the extent of engagement and which partners are engaged and when, there is growing consensus that community engagement is a fundamental guiding principal of the field and that engagement early and often with partners will enhance the likelihood of successful uptake and delivery of EBIs. From an equity perspective, we must prioritize building partnerships and bridges for implementation with local communities and community organizations, particularly those with limited resources that face structural barriers to implementation due to ongoing systemic racism, the legacy of residential segregation, and entrenched policies that benefit some groups and harm others.

If we are not centering the voices and experiences of community partners and practitioners where we are trying to implement, it is not a surprise that it is challenging to implement and sustain EBIs, particularly in communities and settings that experience numerous challenges to achieving health. There are multiple reasons why this engagement is critical in IS, particularly from a health equity perspective (Shelton et al., 2023), including (1) engagement can enhance the fit, relevance, feasibility, appropriateness, and acceptability of EBIs by gaining insights from partners and practitioners in the settings where implementation will occur; (2) it can provide opportunities for communities to identify relevant solutions to overcome implementation challenges, which will help build ownership and enhance trust and trustworthiness; (3) it can enhance sustainability of EBIs and more long-term community capacity by building off of local strengths and embracing sociocultural context; and (4) grounding implementation efforts with input from partners in these settings will help identify challenges, resources, and supports needed to enhance more equitable uptake and delivery.

It is also important to recognize that engagement does not ensure equity in processes or outcomes, and there can be challenges in making progress towards equity in the context of community-academic partnerships (Adkins-Jackson et al., 2022), including in IS. For example, this work requires shifts in resources, power, and decision-making allocation that our grant structures and academic models have not traditionally supported and will require fundamental shifts (e.g., support community members/organizations to co-lead grants; pay community partners equitably for their time; make researchers more accountable to the sustainability of partnerships; provide flexible, longer-term funding mechanisms; Carter-Edwards et al., 2021). There are steps we can take in our own research and at our institutions that work towards more equitable processes, including (1) asking at all phases of research and decision-making who is and is not at the table and take action to remedy this to be more accountable in reach and representation; (2) working with partners to define and operationalize health equity and equity-related goals, priorities, and outcomes in a way that is meaningful and reflective of community priorities; and (3) advocating at our institutions for minimizing administrative barriers that impede equitable partner engagement and resource allocation.

What would it mean to operationalize community engagement with a focus on equity in the context of prevention science and public health? Here, we see important opportunities for strengthening community capacity and partnerships across a range of community and multi-sector settings that matter for health and are central to the ecology of trusted community norms and daily life and have strong potential for enhancing the reach and sustainability of implementation efforts. From a prevention perspective, prioritizing community settings that are vetted and trusted institutions and identifying trusted and trustworthy messengers and champions for implementation has the potential to enhance bi-directional learning and address multiple, intersecting social needs (housing, food security) and health inequities (Kreuter et al., 2021). Thus, while there is value in continuing to partner with health-related non-profits, departments of health, and safety-net health settings, there is also value in partnering with other community settings like educational settings (schools, early childhood centers), worksites, social service and community-based organizations, local government agencies (housing, transportation), and faith-based organizations for implementation and identifying opportunities to better bridge community-clinical linkages (e.g., through community health workers).

There are excellent resources and methods for engagement that can be used to inform and align engagement efforts and build the empirical evidence for engaging for equity in IS (DICE methods at https://dicemethods.org/; Engage for Equity at engageforequity.org) and work we need to do at our institutions to ensure we understand historical and existing community-academic partnerships. There is guidance around best practices for equitable engagement and co-creation in IS and guiding questions for us to reflect on as we apply community engagement principles in IS (Pérez Jolles et al., 2022; Shelton et al., 2023; Shelton, Adsul, Oh, et al., 2021). Critical questions remain, including how to enhance partner trust and trustworthiness to support implementation (Table 1).

## Revisit, Build, and Re-imagine the Evidence Base Needed to Promote Health Equity and Have Impact across Multiple Levels

One of the most fundamental challenges to equitable IS is that implementation efforts are commonly initiated with the assumption that there already is an EBI that has been tested and is effective in improving health or behavior change. However, as noted in prior work (Brownson et al., 2022), due in part to historical reliance on the RCT as the “gold standard” in determining whether something “works” and is evidence-based (which has narrowly defined eligibility criteria and limited generalizability at the individual and setting levels), many EBIs have not been developed with/for or evaluated among populations and settings experiencing inequities. As such, it is not a surprise it is challenging to implement EBIs among groups and settings that they were not designed for and experience different contexts and resource challenges that impact both health and implementation. Community partners/practitioners must be engaged earlier along the translational continuum (e.g., in EBI development and evaluation); this will help address key gaps in our existing evidence base and help ensure that issues that impact effectiveness and implementation are considered from the start.

There is great value in developing and testing interventions in the settings and populations in which they will be evaluated (Beidas et al., 2023). We believe that there are cases (especially in the context of striking inequities) where we must consider the consequences and ethics of not taking action and the value of implementing without “perfect” evidence or efficacy trials (Brownson et al., 2022). We see a critical role for Hybrid I trials as we build the evidence base for interventions that address health equity. Hybrid I trials consider real-world effectiveness and implementation of EBIs early in the evaluation process (Curran et al., 2022). Optimization trials (Guastaferro & Collins, 2021) are also useful and underutilized as a potential approach to understand which intervention components are effective and cost-effective across settings and populations with variable resources.

As we have highlighted (Brownson et al., 2022), this is a timely opportunity for our field to reflect deeply on limitations of our evidence base and the extent to which it has focused on EBIs to promote equity, especially as there has been a paucity of EBIs meaningfully attending to SDOH and equity. This requires that we consider what is valued and “counts” as evidence in our scientific paradigm and for whom an “evidence-based,” as well as reflexivity regarding the values and biases we bring as individual researchers in selecting EBIs. It requires that as we are identifying potential EBIs (ideally with partners/practitioners in those settings), we need to consider where and among who the EBI was tested and the extent to which it may address or acknowledge structural factors and SDOH that impact equitable outcomes. To enhance the impact of IS towards equity, we have the opportunity to prioritize multi-level EBIs that have been co-created with partners, address SDOH, and operate at more upstream levels (not just at the individual level) in multi-sector and community settings.

We must also begin to build the evidence base of interventions that have not historically been perceived as “health” interventions, but have value in addressing health inequities and underlying SDOH. For example, investing in interventions that improve the quality and appearance of abandoned housing has been found to have significant impact on reducing gun violence in Black communities (South et al., 2023). IS frameworks can guide the development of equity-focused interventions with implementation in mind from the start, including the Transcreation Framework which focuses on co-creation and community engagement to elevate community priorities and address inequities (Nápoles & Stewart, 2018). As another example, the CDC Prevention Research Centers Program plays a key role in working with a broad range of community partners to accelerate the dissemination and implementation of EBIs to advance health equity.

There has been a long tradition of elevating “practice-based evidence” to inform intervention and implementation efforts (Green, 2008), and we see this as having promise for equitable impact. We encourage researchers to move away from a strict hierarchy of evidence towards a typology that values different kinds of evidence (Brownson et al., 2022) including community-defined and community-aligned evidence that builds off of existing community strengths. For example, we can track the health benefits from the implementation of a living wage and of programs developed in community settings that are already being delivered but not widely evaluated. Creating greater and more sustainable impact will require implementing EBIs that address key levers, dismantle entrenched health and social inequities, and center and uplift the voices of communities. In addition to growing and expanding our evidence base for equity, it is also important that we understand the impact (on health equity and equitable implementation) of planned adaptations to improve fit with local context and culture. Planned adaptations of EBIs to address SDOH, social needs, or other community priorities can enhance ownership, engagement, cultural appropriateness, and feasibility (e.g., addressing social needs synergistically with cancer screening). Approaches like user-centered design (Dopp et al., 2020) and implementation mapping (Fernandez et al., 2019) can inform equity-focused adaptations of EBIs and enhance fit with needs and context. Key questions remain, including how to balance health and social needs in selecting, developing, adapting, and implementing EBIs (Table 1).

## Prioritize and Elevate a Focus on Policy Development, Dissemination, and Implementation Central to Addressing Equity

Policy should be a central focus of implementation research and efforts focused on advancing health equity. Policies, both small p (organizational policy) and big P (laws, administrative regulations) have profound effects on population health and health equity (Purtle et al., 2023). Policy is a tremendous lever at upstream and local levels that shapes equitable or inequitable access to resources and opportunities that matter for both population health and health equity. It is the political and social context in which health-promoting resources are facilitated or impeded in relation to all key SDOH, including housing, neighborhoods, safety, physical environments, education, economic stability, and healthcare access (e.g., the contexts in which people live, work, and play). Policies influence intersecting, multi-sector resources and practices that can inhibit or hinder health and can shape, reinforce, reduce, or exacerbate health inequities. Importantly, policies are not one size fits all in terms of their impact on equity, and some policies may be more harmful than others for certain groups (e.g., criminal justice policies disproportionately harm Black communities in the USA).

There are multiple aspects of policy-focused research and efforts in IS that have relevance for addressing equity. Policy dissemination research seeks to understand how research evidence can be most effectively communicated to policymakers and integrated into policymaking processes; and policy implementation research seeks to understanding how the roll out of polices can be optimized to maximize health benefits (Hoagwood et al., 2020). The field of policy research in IS is underdeveloped in general (Emmons & Chambers, 2021; Purtle et al., 2016), with even lesser focus on equity-focused policy. Important questions remain as the science of policy dissemination and implementation advances, for example, which dissemination strategies and messaging are effective in facilitating the uptake of equity-focused policies among policymakers (Purtle et al., 2020).

A starting point for policy in IS is the evidence base for action and the effects of implementing the evidence-informed policy. We need a clearer understanding of the effects of various policies on equity because not all policies have equitable reach, uptake, and impact in systemically marginalized communities (Emmons & Chambers, 2021). For example, in a policy-focused umbrella review, Thomson and colleagues studied a wide range of policy approaches across multiple public health areas, showing that while most policies were shown to either improve inequities or were neutral toward inequities, some appear to increase inequities (e.g., low emission zones in cities; Thomson et al., 2018). Building on the Health in All Policies movement (Puska, 2007), Equity in All Policies framing places equity as a primary consideration, not merely one of many considerations. Further, more effort is needed up front in developing policies to ensure that they are evidence-based and reflect the experiences, needs, and priorities of communities experiencing inequities. Equity-focused policy implementation research starts with organizing study questions and elements in ways that fully address barriers to policy progress among socially disadvantaged groups. Despite having over 100 IS theories and frameworks, few specifically focus on policy equity (Crable et al., 2022). Here, lessons can be learned about theories and frameworks from other fields. For example, Zengarini and colleagues applied tenets from Kingdon’s theory (Kingdon, 2010) from political science to co-create policy actions focused on intersectoral actions to address social inequities (Zengarini et al., 2021).

There are related measurement needs for evaluating policy progress. While policy surveillance systems are generally under-developed, specific attention is needed on equity-focused surveillance where data from multiple sectors outside of health (e.g., education, economic development, criminal justice). Several public health systems have begun to develop these policy-relevant data (e.g., Indiana and Seattle King County). Policy implementation is dynamic and complex, and the settings, jurisdictions, and communities where policies delivered have varying levels of resources, infrastructure, competing demands/priorities, and staff shortages which can exacerbate inequities. Additionally, there are changing priorities in the policy landscape and uneven delivery, regulation, monitoring, and enforcement of policies across diverse populations and settings. Multiple gaps in delivery across the policy implementation cascade and inequities in delivery and health impact can arise at every stage, and there is the potential for negative unintended consequences that compound health inequities (e.g., financial costs, mistrust) (see Crable et al., 2022 for guidance on assessing these consequences). Thus, ongoing evaluation and tracking of policies and their impacts on equity and inequities over time is essential and may require removing policies that are harmful or adapting policies over time to maximize benefits (Oh et al., 2021). This requires attention to monitoring and tracking equity in policy effectiveness and uneven implementation over time and consideration of gaps and inequities in policy enforcement/regulation (e.g., where and why do inequities arise across sub-populations, settings, jurisdictions?). Key questions remain (Table 1), including how to optimize equitable policy rollout to maximize impact in systemically marginalized communities.

## Be Agile, Responsive and Adaptive in Application of Frameworks, Processes, and Methods to Enhance the Impact of Implementation Science

It is well documented that the translation to practice is lengthy and moves slowly, which causes challenges to being able to make progress towards equity in a timely manner (Brownson et al., 2023). Producing data that are irrelevant or obsolete by the time the research is completed can serve as an impediment to community-academic partnerships that have pressing needs that do not align with academic timelines. To enhance impact and build the trustworthiness and value of IS to community partners and practitioners, it is important for the field to become more relevant, rapid, and iterative and fit the needs and timeline of partners with limited resources (Riley et al., 2013). The COVID-19 pandemic and social crises have amplified the need for accelerated application of relevant evidence into practice, and there is increasing accountability of community partners and the public in how research is being used to inform community action (Eisman et al., 2022).

Implementation speed is another critically important but understudied area that we must prioritize in order to enhance equitable impact of our science. Proctor and colleagues have defined implementation speed as the “speed of moving from synthesized recommendations based on actionable evidence (e.g., guideline) warranting implementation to the point at which that evidence is identifiable as being used in standard practice, where contextually-appropriate” and have proposed the Framework to Assess Speed of Translation (FAST) framework to advance research and impact in this area (Proctor et al., 2022, p. 108). While inequities in the distribution and speed of EBIs has been apparent for many medical and public health interventions, with time to implementation particularly lengthy in socially disadvantaged communities, COVID-19 made strikingly visible this inequity, with the speed of vaccine distribution and uptake varying significantly across communities by racial/ethnic and socioeconomic characteristics (Jean-Jacques & Bauchner, 2021).

Similar patterns and inequities in health and implementation have been seen in the context of other health issues (e.g., opioid epidemic), with EBIs less readily deployed in the disadvantaged communities most in need. Speed may be particularly important where need is greatest and gaps are largest, given sufficient community demand, low-risks, and sufficient evidence. There are also tensions in accelerating the speed of translation with the goal of equity, as it takes time to understand community priorities and build trusted partnerships that can’t be rushed. At the same time, community/academic partnerships are hindered by the slow pace and the limited impact, relevance, and dissemination of research to communities. Explicit focus is needed on translation of EBIs to enhance relevance, particularly in systemically marginalized communities. Riley and colleagues proposed a model to help enhance and speed of usefulness of research that is relevant to health equity, including early and ongoing partner involvement to enhance fit and relevance, streamlining grant announcements and review process, and planning for rapid and iterative dissemination, implementation, and analyses, resulting in faster availability of data for decision-making (Riley et al., 2013). For example, hybrid designs and pragmatic designs that weigh considerations of internal and external validity are promising approaches for shortening the time to translation, as are more rapid analytic approaches (e.g., rapid qualitative approaches; Ramanadhan et al., 2021).Our science needs to be adaptive and rapid to be responsive to community priorities, dynamic context, and learnings from practice-based settings. This requires that we not only adapt interventions and strategies focused on promoting equity as we learn what is and is not working, but that we refine frameworks, theories, and models based on learnings from practice and partner engagement. Examples have included refining implementation frameworks and making adaptations in their application to have more explicit focus on health equity, including the Racism-conscious adaptation of the Consolidated Framework for Implementation Research (Allen et al., 2021). If we are using frameworks that do not consider contextual factors that are important for addressing health equity and are relevant to the settings and populations (e.g., social, structural, and community context), it is essential that we refine our frameworks to reflect these equity-specific determinants.

There are several examples of a rapid, learning health systems approach where we can be more iterative in applying what we learn for equity-focused IS. This includes iterative application of the RE-AIM model engaging partners with multiple perspectives to understand the relative importance and progress on implementation outcomes and to inform real-time adaptations during implementation and develop consensus-based strategies (Glasgow et al., 2020). To avoid being reactive, there is a need for more infrastructure and interactive tools to guide planning and real-time data return in public health, social service, and community settings (e.g., data dashboards; see the HEALing Communities Study; Wu et al., 2020). Researchers have examined how factors like poverty and discrimination create barriers to EBI use and how adaptations to culture, language, literacy, or delivery system characteristics can address gaps in implementation for lower-resource settings (Aschbrenner et al., 2021). Engaged data-driven approaches may be useful to help identify and prioritize health equity gaps and guide adaptations to enhance equity. For example, processes to support stakeholder and equity data-driven implementation have been used in community health centers to obtain healthcare data to identify patient groups experiencing gaps in use of EBIs and rapidly adapt them to enhance access and equitable using a rapid cycle testing approach (Aschbrenner et al., 2022).

At a broader level, an example of a research network that has prioritized rapid approaches is the NCI-funded Implementation Science Centers, which were designed to be responsive to emerging priorities of community partners and address dynamic multilevel context in learning-oriented approaches that leverage partner expertise (Oh et al., 2023). The centers emphasize pragmatic designs and the rapid generation and dissemination of results to partners to help translate findings into action. The integration of pilot studies enables responsivity to partners, dynamic context, and testing of innovative strategies in diverse settings with room for rapid learning and adaptation as needed. Important issues remain, including how soon we should act on evidence in systemically marginalized communities that experience well-placed mistrust and distrust of research (Table 1).

## Identify and Redefine Meaningful Metrics for Equity and Impact in Implementation Science

We enter IS to make a positive impact on society and health equity. As researchers, we generally do an excellent job of tracking impacts of our scholarship in ways relevant for academia (e.g., publications, citation rates, grants)—these metrics have limited utility in demonstrating broader, real-world impacts, including equity among minoritized communities. A stronger focus is needed on documenting real-world impacts of IS, including health equity. In a systematic review, Alla et al. (2017) identified four domains of how impacts are defined (in order of frequency) that may be useful for IS to consider, including (1) contributions or area of focus (e.g., economy, environment, services); (2) avenues or processes (e.g., effects on knowledge, creating new products); (3) change (e.g., specific benefits, harms, positive returns); and (4) levels (e.g., individual, local, national).

In the UK and the USA, several frameworks have been developed to document impacts beyond the usual academic metrics. In the UK, the Research Excellence Framework (REF) is a national evaluation system for assessing the quality of research in institutions of higher education. In part, REF provides recognition to institutions and researchers that have built on strong research to deliver demonstrable benefits to the economy, society, public policy, culture, or quality of life (Jensen et al., 2022). It recognizes that real-world impacts may occur 10–15 years following the original research and provides useful tools for telling the story of research impacts (Tilley et al., 2018). In the USA, the Translational Science Benefits Model (TSBM) provides a framework and benchmarks to measure the impact of scientific discoveries beyond traditional metrics, including (1) clinical and medical benefits; (2) community and public health benefits; (3) economic benefits; and (4) policy and legislative benefits (Luke et al., 2018). Using the TSBM, both quantitative and qualitative approaches can be used to assess impacts. As we apply these frameworks more systematically, it is critical that we deepen and make more explicit the equity focus and impacts for all domains, including narratives and metrics around benefits and unintended consequences.

**Fig. 1:**
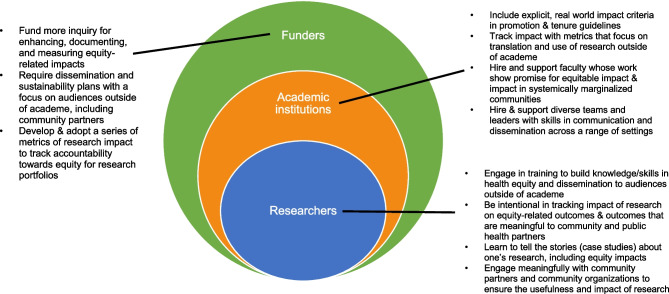
Sample actions for researchers, academic research institutions, and funding agencies to increase research impact and equity in Implementation Science

Regardless of the impact framework being applied, it is useful to consider potential actions that researchers, academic institutions, and funders can take to enhance equity and impact in IS (see example actions, Fig. 1). Actions at these three levels should be informed by community needs and input from key partners; taken together, actions in these nested levels are likely to enhance and better document the tangible benefits of IS. There is a need for quantitative metrics, as well as qualitative narratives to record impacts on overall health, systems change, and progress towards health equity. To date, the most common impacts identified in case studies are influencing professional guidelines, informing policy change, and changing clinical practice (Greenhalgh & Fahy, 2015).

As we start to demonstrate the impacts we have with our science, we must make clear the metrics we use to track improvements in health equity and how these are operationalized. Often progress is made towards equity by tracking or measuring reductions or improvements in health outcomes, behaviors, healthcare access, quality of life, or reductions or exacerbation of disparities between groups (e.g., differences across racial/ethnic groups in cancer mortality). In IS, there are additional levels of progress towards equity that are important to track and be accountable to. For example, we often start with the “research-to-practice” gap as the basis or rationale for our implementation efforts, which are often assessed as low adoption, uptake, and implementation of an EBI within or across settings. However, that research to practice gap is often greater in systemically marginalized communities or settings due to historical structural and social drivers which impact the allocation of resources, including EBIs. Thus, as part of our tracking of equity in IS, we should strive to capture the extent to which historical or ongoing implementation efforts have reduced or exacerbated the research to practice gap across populations and settings that experience varying levels of structural barriers. For equity metrics we track in IS, there are benefits to trying to align these with accountability metrics that are already in place at organizational, systems, and policy levels (e.g., building equity metrics in an existing dashboard for tracking improvements to the transportation system). An additional metric for tracking progress towards health equity in IS is examining the extent to which there is equitable adoption, implementation, and sustainability of EBIs and strategies across diverse settings and populations. We may look to the literature, existing local data or data dashboards, or formative research with partners to identify what the most striking dimension(s) of inequities are that we are trying to address and should be prioritized. Such inequities may relate to salient setting characteristics (e.g., FQHC or rural areas) or to population characteristics (e.g., income, race, ethnicity, language).

Health equity is not one size fits all and may look different in its definition according to where you are based; for example, within a FQHC or non-profit serving homeless youth, equity may be central to all service delivery and implementation, whereas in an academic medical center, it may be one of many aspects of implementation. Regardless of whether health equity is the primary focus of a particularly implementation study, there is value in tracking whether there is equitable delivery and uptake across all settings and groups. It is critical to work with local partners and practitioners to determine how health equity is being defined and what is meaningful to partners in that setting and pragmatic to assess and address. Models like RE-AIM and the RE-AIM extension for equity and sustainability can be used to help identify when and where the most pressing inequities arise or are exacerbated across implementation phases (e.g., reach, adoption, implementation, effectiveness, maintenance; Glasgow et al., 2019; Shelton et al., 2020). These tools can help specify where and why these gaps exist and can help inform needed adaptations and areas where resource investments should be prioritized if implementation is uneven. This requires that we be explicit about the extent to which inequities are exacerbated through implementation processes and strategies (i.e., varying health benefits, implementation, and/or unintended consequences across sub-groups). Future research should assess the extent to which our strategies to address these gaps and inequities along the implementation cascade are effective and the mechanisms through which they operate that are specific to equity considerations (e.g., do strategies reduce stigma and improve trust?) (Table 1).

## Disseminate Scientific Evidence and Research Findings to Diverse Partners

From an equity perspective, to have the intended impacts outside of academe, findings from implementation research need to be disseminated to a set of relevant audiences including community partners, practitioners, and policymakers. Effective and efficient dissemination is informed by three bodies of scholarship: designing for dissemination (D4D) (Brownson et al., 2013; Kwan et al., 2022), diffusion theory (Rogers, 2003), and communication science. We will also briefly review approaches for framing and disseminating health equity benefits to various audiences.

Designing for dissemination is a phased process defined as *the process of ensuring that the products of research are developed to match the contextual characteristics of the target audience and setting* (Kwan et al., 2022). For health equity, this requires that they match the needs and priorities of settings and populations experiencing health and social inequities. Effective D4D begins early in the research process with a conceptualization phase to determine the need and demand for a solution to a health problem (the pull). Next comes a design phase to determine dissemination products and their packaging for delivery. In an active dissemination phase, a team makes use of systems and infrastructure to disseminate the product package to intended audiences. In the final impact phase, tracking should include a range of metrics, including explicitly tracking effects on health equity.

Other essential dissemination principles are from diffusion of innovations theory (Rogers, 2003). For example, a subset of adopters of a new practice are opinion leaders who often have a strong influence on others (Miech et al., 2018). Attributes of the innovation (e.g., the EBI) are important—it needs to show advantage over existing practices, it can be tried on a small scale, and its costs matter (Dobbins et al., 2002). As we build an evidence base for equitable dissemination, we must spend time understanding who are the influential and trusted opinion leaders in these priority settings; we must also understand the characteristics of EBIs that are of value to key partners and will influence the speed and extent of adoption.

Building on concepts from communication science, it is important to apply principles of audience segmentation, framing, and message tailoring. Audience research studies provide an empirical foundation to inform the design and distribution of dissemination materials (Slater et al., 2006). When disseminating an implementation product to an audience outside of academe, it is key to describe audience characteristics, potentially useful messages, and the channels (how to reach the audience) that are most likely to be effective. Examples are shown in Table 2. For policy audiences, message framing may involve a gain vs. loss mindset (dollars saved vs. lives lost), how effective messages are perceived (unbiased, credible), how to deliver messages (appropriately packaged, understandable), and timing (available when needed) (Morshed et al., 2017; Purtle et al., 2020).

**Table 2:** Planning matrix and considerations for audience segmentation for more impactful dissemination efforts and messaging

**Segment**	**Relevant characteristics**	**Messages**	**Channels**
Public health practitioners	• High commitment to health • Wide range of professional backgrounds • Access to summaries of evidence but often not the original research • Long-term horizon for outcomes	• Make a difference in society • Improve health equity • Enhance resources	• Leadership meetings • Professional associations • Brief summaries of evidence
Clinical practitioners	• High commitment to health • Narrow range of professional backgrounds • Time urgency	• Improve patient care • Improve health equity	• Journal articles • Professional associations • Professional conferences • Brief summaries of evidence
Policymakers (elected officials and street-level bureaucrats)	• Variable commitment to health (often limited knowledge across many issues) • Wide range of professional backgrounds • Short-term horizon for outcomes	• Serve constituents • Create return on investment • Get re-elected • Congruence of outcomes with strategic plans/agency aims	• Real-world stories • Brief summaries of evidence • Delivery of messages by opinion leaders
Community members and community partners	• Variable commitment to health • Values different types of “knowledge” and “evidence” regarding health • Impacted by personal or familial experiences as patients • Wide range of professional backgrounds • Shorter term horizon for outcomes	• Makes a difference in personal health/health of community • Provides tangible benefits or relevance to self, family, community • Is valued service or resource to community • Will not incur high costs or financial burden	• Local media channels • Real-world stories • Culturally appropriate media (local papers, radio) • Delivery of messages by trusted local community leaders • Cost-effectiveness and economic impacts of interventions

Dissemination to enhance health equity should reflect the needs and contexts of specific groups and will require new thinking and approaches. To improve dissemination processes, researchers should engage with equity-focused partners (including community members) through the research process. These partners can help inform approaches for audience segmentation, refinement of messages, and tracking relevant outcomes from dissemination. The context and products of dissemination also need to account for the potential for implicit biases, harmful institutionalized practices, and negative attitudes towards groups experiencing inequities (Farrer et al., 2015; Purtle et al., 2023). Critical questions remain, including, how to support equitable dissemination of research findings for all partners engaged (Table 1).

## Extend Our Focus to the Science of De-implementation, Mis-implementation, and Sustainability Which are Central to Impacting Equity

Over its brief history, the main focus of IS has been on the initial implementation of EBIs. More recently, there has been a growing focus on the over-use and mis-use of EBIs (de-implementation or mis-implementation). De-implementation refers to the reduction or discontinuation of interventions that are low-value (i.e., inappropriate, ineffective, or potentially harmful; McKay et al., 2018). Attempts at de-implementing use of familiar interventions run into entrenched beliefs that “more is better” (Schlesinger & Grob, 2017). Mis-implementation is a process where effective interventions are ended or ineffective interventions are continued in public health settings (i.e., evidence-based decision making is not occurring; Brownson et al., 2015). Mis-implementation is far too common. For example, local health department staff reported that between 30 and 42% of programs are discontinued when they should continue and between 16 and 29% of programs continue that should have ended (i.e., continuing ineffective programs; Allen et al., 2020; Brownson et al., 2015).

Both de-implementation and mis-implementation have profound health equity implications. For example, Black and Latino patients are more likely to receive low value health care for multiple services (Schpero et al., 2017). In low resource settings, it is critical to carefully consider the contextual factors (e.g., history of racism, inequitable policies that benefit some and harm others) when seeking remedies for overuse or underuse of interventions (McKay et al., 2018). Between 10 and 30% of US healthcare spending is due to overuse of low value care (Brownlee et al., 2014). When this magnitude of overuse occurs, fewer resources are available for patients most in need. A richer understanding of de-implementation and mis-implementation will help us better allocate limited resources to be used more efficiently. Additionally, advancing the science of de-implementation will also enable understanding of how to remove and dismantle policies and practices that are institutionalized in systems and organizations and are harmful in contributing to health inequities.

Sustainability of EBIs is another critically important but understudied area in IS that has critical implications for health equity. Investing limited resources and time in implementation of EBIs that are then not continued or maintained can result in wariness and frustration on the part of practitioners and partners and may reinforce the lack of trustworthiness of our research institutions (Shelton & Nathan, 2022). This may harm community-academic partnerships and prevent communities or organizations from partnering in future implementation efforts. As such, it is important that we advance the science and invest resources in how to successfully sustain EBIs (particularly in lower-resource settings) and understand the contextual factors and strategies that can better support continued delivery in a pragmatic and cost-effective way. Communities and settings that face structural barriers to health are less likely to receive the benefits of long-term investments in sustaining EBIs and should be priority areas for advancing the science of sustainability. To make the case for investing in the sustainability of EBIs, it is critical that we prioritize including costing and economic evaluation to enhance understanding of the value of EBIs (Gold et al., 2022). Many key questions remain (see Table 1).

## Conclusion and Discussion

We are at a critical inflection point for reflecting on gaps and opportunities to enhance the impact of our science. EBIs will not have transformative impact on the health of many communities if they are not disseminated, adopted, and widely used in a timely manner and both routinely and equitably integrated into public health infrastructure and healthcare systems. We believe that the application of IS is key to accelerate and track progress towards health equity and equitable impact of prevention policies and programs at scale. We recognize that our calls to action laid out here will require time, reflection, intentionality, and humility in how we conduct science and engage partners and require larger shifts and investments in how we approach grants and partnerships. Transforming health systems and having funding and a political landscape that upholds this vision is part of the broader context that supports or inhibits equitable implementation; major shifts towards equity in our broader societal and policy context are needed to support these changes in a sustainable way.

Within each of the priority areas we have outlined, there are practical steps we can take now to advance the impact and science of equity-focused implementation. First, we must make health equity explicit and be clear on what our definition is and how we are making equity foundational in framing research questions, selecting methods and frameworks, deploying resources, and disseminating findings. Second, we must be attuned to what health equity means in the context where we are working and focus on both health-equity processes and approaches (the how), in addition to health equity outcomes. Third, we must recognize and advance the science on the wide range of dimensions through which inequities exist, including setting characteristics (e.g. geography), and consider how they intersect with other social dimensions (e.g., race, age, income, language, gender). Finally, it is important to reflect on and consider what equitable and meaningful partnership and engagement looks like in public health and community settings to facilitate reach, build trust, share power, support the needs of diverse communities, and address barriers to equitable implementation.

It is important to recognize that the “research-to-practice” gap in IS is strongly shaped by broader social inequities and structural drivers and forces that we have the opportunity to help identify and dismantle. If we want to apply IS to have greater impact and make progress on equity, we need to do a better job at recognizing and disrupting the downstream effects of this broader historical context that continues to reify and reinforce health inequities in the healthcare systems, public health organizations, and communities with which we partner. Creating impact requires prioritizing EBIs and strategies that address and minimize the downstream effects of structural and social determinants of health. Additionally, if we want to make big shifts towards equity instead of chipping away slowly and in compartmentalized silos towards health inequities, we must make the conscious decision to prioritize and partner with those settings and communities that have experienced disinvestments and have been excluded and deprioritized intentionally or unintentionally in our implementation efforts.

In conclusion, this paper does not seek to raise or answer all of the important questions in the field, with many pressing questions remaining, including as follows: Who should lead and conduct equity-focused IS? What are the competencies and partnerships necessary to conduct equity-focused IS? How do we bring more under-represented individuals into IS? Who sets the research agenda and questions that are prioritized and funded in IS? What are the unintended consequences of implementation research that may worsen or exacerbate inequities? However, it is our hope that this paper helps to further spark a commitment in our field to conduct and lead more equitable partnerships and research that will have greater and more far-reaching beneficial impacts for all.

## Declarations

### Disclaimer

Conclusions in this paper are the authors and do not necessarily represent the official positions of these agencies.
